# Intervessel pit membrane thickness best explains variation in embolism resistance amongst stems of *Arabidopsis thaliana* accessions

**DOI:** 10.1093/aob/mcaa196

**Published:** 2020-11-20

**Authors:** Ajaree Thonglim, Sylvain Delzon, Maximilian Larter, Omid Karami, Arezoo Rahimi, Remko Offringa, Joost J B Keurentjes, Salma Balazadeh, Erik Smets, Frederic Lens

**Affiliations:** 1 Naturalis Biodiversity Center, Research Group Functional Traits, RA Leiden, The Netherlands; 2 BIOGECO INRA, Université Bordeaux, Pessac, France; 3 Plant Developmental Genetics, Institute of Biology Leiden, Leiden University, BE Leiden, the Netherlands; 4 Laboratory of Genetics, Wageningen University, Droevendaalsesteeg, PB Wageningen, The Netherlands

**Keywords:** *Arabidopsis thaliana*, embolism resistance, herbaceous species, intervessel pit membrane, lignification, stem anatomy, xylem hydraulics

## Abstract

**Background and Aims:**

The ability to avoid drought-induced embolisms in the xylem is one of the essential traits for plants to survive periods of water shortage. Over the past three decades, hydraulic studies have been focusing on trees, which limits our ability to understand how herbs tolerate drought. Here we investigate the embolism resistance in inflorescence stems of four *Arabidopsis thaliana* accessions that differ in growth form and drought response. We assess functional traits underlying the variation in embolism resistance amongst the accessions studied using detailed anatomical observations.

**Methods:**

Vulnerability to xylem embolism was evaluated via vulnerability curves using the centrifuge technique and linked with detailed anatomical observations in stems using light microscopy and transmission electron microscopy.

**Key Results:**

The data show significant differences in stem *P*_50,_ varying 2-fold from −1.58 MPa in the Cape Verde Island accession to −3.07 MPa in the woody *soc1 ful* double mutant. Out of all the anatomical traits measured, intervessel pit membrane thickness (*T*_PM_) best explains the differences in *P*_50_, as well as *P*_12_ and *P*_88_. The association between embolism resistance and *T*_PM_ can be functionally explained by the air-seeding hypothesis. There is no evidence that the correlation between increased woodiness and increased embolism resistance is directly related to functional aspects. However, we found that increased woodiness is strongly linked to other lignification characters, explaining why mechanical stem reinforcement is indirectly related to increased embolism resistance.

**Conclusions:**

The woodier or more lignified accessions are more resistant to embolism than the herbaceous accessions, confirming the link between increased stem lignification and increased embolism resistance, as also observed in other lineages. Intervessel pit membrane thickness and, to a lesser extent, theoretical vessel implosion resistance and vessel wall thickness are the missing functional links between stem lignification and embolism resistance.

## INTRODUCTION

Long-distance water transport in the xylem connecting roots to leaves is essential for plant survival and distribution (Sperry, 2003; Brodribb, 2009; Lucas *et al.*, 2013; Lens *et al.*, 2016; Trueba *et al.*, 2017; Choat *et al.*, 2018; Brodribb *et al.*, 2020). Plants have developed an ingenious system to transport water upwards against gravity by a largely passive mechanism that is driven by a difference in negative xylem pressure created in the leaf mesophyll cell walls, known as the cohesion-tension theory (Dixon and Joly, 1895; Pickard, 1981; Brown, 2013). However, this negative or subatmospheric pressure inside the water-conducting xylem conduits puts water in a metastable liquid state, making it vulnerable to heterogeneous cavitation: the transition from liquid water to vapour by spontaneous destabilization of the hydrogen bonds between water molecules at nucleating sites (Steudle, 2001; Wheeler and Stroock, 2008; Brown, 2013; Venturas *et al.*, 2017). Under drought stress conditions, the xylem pressure becomes more negative, thereby increasing the risk of tiny vapour bubbles enlarging into a large embolism that blocks the water transport inside a conduit (Sperry and Tyree, 1988; Tyree and Zimmermann, 2002; Cochard, 2006). This embolized conduit can then cause gas bubbles to spread towards adjacent water-filled conduits via tiny pores in the interconduit pit membranes, a process called air-seeding. Air-seeding may lead to a rapid spread of drought-induced embolism throughout the plant, giving rise to hydraulic failure, i.e. a catastrophic loss of xylem hydraulic conductance, ultimately causing plant death (Brodribb and Cochard, 2009; Allen *et al.*, 2010; Urli *et al.*, 2013; Anderegg *et al.*, 2016; Brodribb *et al*., 2016, 2020; Adams *et al.*, 2017; Kaack *et al.*, 2019; Zhang *et al.*, 2020). Acquiring a sufficient level of embolism resistance, therefore, represents one of the most essential adaptations for plant survival under drought conditions, along with other strategies such as reduced water loss, increased water storage or root depth (Lens *et al.*, 2013; Gleason *et al.*, 2014; Martin-StPaul *et al.*, 2017; Billon *et al.*, 2020).

The relationship between the decline in hydraulic conductivity due to embolism and xylem pressure is plotted in a so-called vulnerability curve, from which the pressure inducing 50 % loss of hydraulic conductivity (*P*_50_) – the often-cited proxy for drought tolerance – is derived (Maherali *et al.*, 2004; Choat *et al.*, 2012; Venturas *et al.*, 2017). Hydraulic studies show a wide range of *P*_50_ across species (from −0.5 to −20 MPa), and species occupying dry habitats are generally more resistant to embolism formation (more negative *P*_50_) than species from wet habitats (Brodribb and Hill, 1999; Choat *et al.*, 2012; Larter *et al.*, 2015; Lens *et al.*, 2016; Trueba *et al.*, 2017). Xylem physiologists have measured *P*_50_ values in stems of over 2000 tree and shrub species. However, hydraulic measurements in herbaceous species are limited to only a few dozen species, despite the fact that a majority of our important food crops are herbs (Stiller and Sperry, 2002; Holloway-Phillips and Brodribb, 2011; Lens *et al*., 2013, 2016; Nolf *et al.*, 2016; Skelton *et al.*, 2017; Ahmad *et al.*, 2018; Dória *et al.*, 2018; Volaire *et al.*, 2018; Bourbia *et al.*, 2020; Corso *et al.*, 2020; Lamarque *et al.*, 2020). Therefore, it is essential to focus more on herb hydraulics and integrate these hydraulic traits in models that predict annual crop yields to consider the effects of drought and heatwave events (Asseng *et al.*, 2015).

In this paper, we focus on the model species *Arabidopsis thaliana*. This small herbaceous species is able to produce a limited amount of wood in the hypocotyl and at the base of the inflorescence stem (Chaffey *et al.*, 2002; Ko *et al.*, 2004; Nieminen *et al.*, 2004; Melzer *et al.*, 2008; Lens *et al.*, 2012). Wood formation can be moderately induced in wild-type accessions by either delaying flowering time under short days (Tixier *et al.*, 2013) or by clipping developing flowers (Chaffey *et al.*, 2002), by applying weights on the inflorescence stem (Ko *et al.*, 2004) or by increasing auxin levels (Agusti *et al.*, 2011; Brackmann *et al.*, 2018). A more extensive wood cylinder can be induced by modifying gene regulation that turns the herbaceous phenotype into a shrubby phenotype (Melzer *et al.*, 2008; Karami *et al.*, 2020), although this woodiness does not extend to the upper parts of the inflorescence stems (Lens *et al.*, 2012). Since increased woodiness or lignification levels in stems have been linked to higher levels of embolism resistance in various plant groups (Lens *et al*., 2013, 2016; Tixier *et al.*, 2013; Dória *et al*., 2018, 2019), we selected three herbaceous wild-type accessions of *A. thaliana* with different growth types and drought responses [Columbia (Col-0), Cape Verde Islands (Cvi) and Shahdara (Sha); Bac-Molenaar *et al.*, 2016; Thoen *et al.*, 2017] and one woody mutant established in the Col-0 background (*soc1 ful* knockout; Melzer *et al.*, 2008) to evaluate this potential correlation more closely. To this end, we applied the Cavitron centrifuge method (Cochard *et al.*, 2013) to compare the xylem embolism resistance of inflorescence stems amongst the four accessions, and assessed which xylem anatomical traits underlie the differences observed in *P*_50_ using detailed anatomical observations with light microscopy (LM) and transmission electron microscopy (TEM). Various hydraulically relevant stem traits were observed, such as the proportion of stem woodiness/lignification, intervessel pit membrane thickness, fibre wall thickness, theoretical vessel implosion index and vessel grouping index (Table 1). We hypothesize that woodier or more lignified *Arabidopsis* stems are more resistant to embolism formation than less lignified stems and that this difference in embolism resistance is functionally driven by intervessel pit membrane thickness.

**Table 1.** List with the anatomical characters measured with reference to their acronyms, definitions, calculations, microscope techniques, and units

Acronym	Definition	Calculation	Number of measurements	Unit	Technique
*A*_F_	Fibre cell area	Area of single xylem fibre in cross-section	Min. 30 fibres	μm^2^	LM
*A*_FL_	Fibre lumen area	Area of single xylem fibre lumen in cross-section	Min. 30 fibres	μm^2^	LM
*A*_FW_	Fibre wall area	*A*_F_ – *A*_FL_ for the same fibre	Min. 30 fibres	μm^2^	LM
*A*_LIG_	Lignified stem area	Total xylem area + fibre caps area + lignified pith cell area in cross-section	9 stems per accession	mm^2^	LM
*A*_PITH_	Pith area	Total pith area in cross-section	9 stems per accession	mm^2^	LM
*A*_S_	Total stem area	Total stem area in cross-section	9 stems per accession	mm^2^	LM
*D*	Diameter of vessels	Equation 3	Min. 50 vessels	μm	LM
D_H_	Hydraulically weighted vessel diameter	Equation 4	Min. 50 vessels	μm	LM
*D*_MAX_	Maximum vessel lumen diameter	Diameter of single vessel	Min. 30 vessels	μm	LM
*D*_PC_	Pit chamber depth	Distance from the relaxed pit membrane to the inner pit aperture	Min. 25 pits	μm	TEM
*P*_FW_F_A_	Proportion of fibre wall area per fibre cell area	*A*_FW_/*A*_F_ for the same fibre; a measure of xylem fibre wall thickness	Min. 30 fibres	–	LM
*P*_LIG_	Proportion of lignified area per total stem area	*A*_LIG_ /*A*_S_	9 stems per accession	–	LM
*T*_PM_	Intervessel pit membrane thickness	Thickness of intervessel pit membrane measured at its thickest point	Min. 25 measurements	μm	TEM
T_V_	Vessel wall thickness	Thickness of a single vessel wall	Min. 30 Vessels	μm	LM
*T*_VW_/*D*_MAX_	Thickness-to-span ratio of vessels	Double intervessel wall thickness divided by the maximum diameter of the largest vessel	Min. 30 measurements	μm	LM
(*T*_VW_/*D*_MAX_)^2^	Theoretical vessel implosion resistance	(*T*_VW_/*D*_MAX_)^2^	Min. 30 measurements	–	LM
*V*_D_	Vessel density	Number of vessels per mm^2^	Min. 5 measurements	No. of vessels/mm^2^	LM
*V*_G_	Vessel grouping index	Ratio of total number of vessels to total number of vessel groupings (including solitary and grouped vessels)	Min. 50 vessel groups	–	LM

## MATERIALS AND METHODS

### Plant material

Three accessions and one woody mutant of *Arabidopsis thaliana* were chosen based on their contrasting growth forms, the difference in drought tolerance and the minimum length of their inflorescence stems: (1) Columbia (Col-0, a direct descendant of Col-1 from Poland and Eastern Germany; Passardi *et al.*, 2007; Somssich, 2019; Koornneef and Meinke, 2010); (2) Shahdara (Sha, native to a low-precipitation area of Shakhdarah valley, Tajikistan; Khurmatov, 1982; Trontin *et al.*, 2011); (3) Cape Verde Islands (Cvi, native to the high-altitude region above 1200 m on Cape Verde Islands; Lobin, 1983; Monda *et al.*, 2011); and (4) a Col-0 accession in which two flowering time control genes, SUPPRESSOR OF OVEREXPRESSION OF CO 1 (SOC1) and FRUITFULL (FUL), are knocked out (*soc1 ful* in the Col-0 background; Melzer *et al.*, 2008). The three wild-type accessions were selected based on the length of their inflorescence stems [at least 30 cm is required for the Cavitron measurements; this exceeds by far the maximum vessel length of Col-0, which reaches only 4 cm according to Tixier *et al.* (2013), to avoid potential open-vessel artefacts (Cochard *et al.*, 2013)], and their differences in drought response (Bac-Molenaar *et al.*, 2016; Thoen *et al.*, 2017) and growth form. The *soc1 ful* knockout was selected as the woody counterpart because of its extended levels of wood formation at the base of the inflorescence stems (Lens *et al.*, 2012). One hundred individuals from three accessions and one double knockout were grown from seeds sown directly in a mixture of soil and sand (4.5:1). After seed germination (10–12 d after sowing), the healthy seedlings were transferred and grown individually in 8-cm-diameter pots in a growth chamber under controlled conditions of 20 °C temperature and 16-h photoperiod, with 100 μmol m^−2^ s^−1^ light intensity. Relative humidity was maintained at 70 %. We synchronized the harvesting time for the four accessions, meaning that each accession was harvested at different ages (55–65 d for wild-type accessions, 80–90 d for *soc1 ful*), depending on the time required for flowering and inflorescence stem development.

### Xylem vulnerability to embolism

*Sample preparation of inflorescence stems*. The plants were harvested, with roots, leaves and flowers still attached, in the growth chamber facilities at the Institute of Biology Leiden (Leiden University, The Netherlands). The basal part of the inflorescence stems of each accession was cut underwater with a sharp razor blade to a length of at least 30 cm, and then immediately wrapped in wet tissues, enclosed in plastic bags and shipped to the PHENOBOIS platform (INRAE, University of Bordeaux, France) for the hydraulic experiments, which were carried out within a week of harvest. Before running the Cavitron centrifuge measurements, the samples were recut underwater to a standard length of 27 cm, after which both ends were trimmed to fit the Cavitron rotor. All siliques, leaves and flowers were removed from the stems just before the measurement.

*Cavitron centrifuge method*. Centrifugal force has been used to induce cavitation in stem segments by lowering the xylem pressure in the middle part of stems during spinning (Cochard, 2002; Cochard *et al.*, 2005). Vulnerability to embolism in the inflorescence stems was measured using ten individuals per vulnerability curve to generate sufficient hydraulic conductivity during the spinning experiment; about ten vulnerability curves per accession were generated. A solution of deionized ultrapure water containing 1 mm CaCl_2_ and 10 mm KCl was used as a reference for the hydraulic conductivity measurements. The theoretically maximum hydraulic conductivity (*K*_max_, m^2^ MPa^−1^ s^−1^) of the ten inflorescence stems was firstly calculated at near-zero MPa (low speed). The xylem pressure was then gradually decreased by −0.2 to −0.4 MPa for each spinning step. The hydraulic conductivities at every rotation speed (*K*) were measured using Cavisoft software (Cavisoft v1.5, University of Bordeaux, France). The percentage loss of hydraulic conductivity (PLC) was computed as:


$$\begin{array}{l} PLC=100\times \left(1\left(K/K_{MAX}\right)\right) \end{array}$$ (1)

The vulnerability curves were constructed and fitted with a sigmoid function (Pammenter and Vander Willigen, 1998) using the NLIN procedure in SAS 9.4 (SAS 9.4; SAS Institute, Cary, NC, USA) following the equation:


$$\begin{array}{l} PLC=100/[1+exp(\left(S/25\right)\times (P-P_{50}))] \end{array}$$ (2)

where *P* is the xylem pressure value used at each step, *P*_50_ represents the xylem pressure inducing 50 % loss of hydraulic conductivity and *S* (% MPa^−1^) is the slope of the vulnerability curve at the inflexion point (*P*_50_).

### Stem anatomy

*Sample preparation.* Since stem anatomy at the basal, more lignified part differs rather considerably compared with the middle part, where the negative pressures were applied during the Cavitron measurements, we made sections from both parts and performed the anatomical observations on the middle stem parts to match anatomy with *P*_50_. From the ten vulnerability curves we generated per accession, we selected three stems for three representative vulnerability curves (nine individuals per accession) for LM, and one stem for three representative vulnerability curves (three individuals per accession) for TEM. The anatomical measurements (Table 1) were carried out using ImageJ (National Institutes of Health, Bethesda, MD, USA) following the recommendations of Scholz *et al.* (2013).

*Light microscopy*. The inflorescence stems were cut into small pieces ~1 cm long and stored in 70 % ethanol. Fixed samples were then infiltrated and embedded in LR-White resin (Hamann *et al.*, 2011). The embedded samples were sectioned using a Leica RM 2265 microtome with disposable tungsten carbon blades (Leica, Eisenmark, Wetzlar, Germany) at a thickness of 4 μm. Subsequently, the sections were heat-fixed onto the slides with 40 % acetone, stained with toluidine blue [1 % (w/v) toluidine blue (VWR Chemicals BDH^®^, Radnor, PA, USA) in 1 % (w/v) borax], rinsed with distilled water, air-dried, and mounted with DPX new-100579 mounting medium (Merck Chemicals, Amsterdam The Netherlands). The anatomical features were observed under a Leica DM2500 light microscope and photographed with a Leica DFC-425 digital camera (Leica microscopes, Wetzlar, Germany). The diameter of vessels (*D*) was calculated as:


$$\begin{array}{l} D=(\sqrt{4A})/\pi \end{array}$$ (3)

where *D* represents the diameter of vessels and *A* is the conduit surface area. The hydraulically weighted vessel diameter (*D*_H_) was calculated based on the diameter of vessels (*D*) following the equation (Tyree and Zimmermann, 2002):


$$\begin{array}{l} D_{H}={\left(\sum D^{4}/N\right)}^{1/4} \end{array}$$ (4)

where *D* is the diameter of vessels measured using eqn (3) and *N* is the number of conduits measured. All the measurements are explained in Table 1.

*Transmission electron microscopy*. After the Cavitron experiment, 1-cm-long pieces from the middle part of the inflorescence stems were immediately collected and fixed in Karnovsky’s fixative for 48 h (Karnovsky, 1965). The samples were cleaned three times in 0.1 m cacodylate buffer, then post-fixed with 1 % buffered osmium tetroxide, rinsed again with buffer solution, stained with 1 % uranyl acetate, and dehydrated in a series of ethanol: 1 % uranyl acetate replacement, with increasing concentration of ethanol (30, 50, 70, 96 %, and twice in ≥99 %). The samples were then infiltrated with Epon 812n (Electron Microscopy Sciences, Hatfield, UK) and placed at 60 °C for 48 h in the oven. The Epon blocks were trimmed to a thickness of 2 μm using a rotary microtome with a glass knife. Subsequently, the cross-sections with many vessel–vessel contact areas were cut into ultrathin sections of 90–95 nm using a Leica EM UC7 ultramicrotome with a diamond knife. The sections were dried and mounted on film-coated copper slot grids with Formvar coating (Agar Scientific, Stansted, UK), and post-stained with uranyl acetate and lead citrate. Ultrastructural observations of intervessel pits were performed and photographed using a JEM-1400 Plus TEM (JEOL, Tokyo, Japan) equipped with an 11-megapixel camera (Quemesa, Olympus). At least 25 relaxed, non-shrunken intervessel pit membranes were selected from three individuals per accession to observe intervessel pit membrane thickness and pit chamber depth (Table 1).

### Statistical analysis

To assess the differences between embolism resistance among the four accessions studied we used general linear models. A Newman–Keuls *post hoc* test was applied to test whether or not embolism resistance (*P*_50_) and anatomical characters differ amongst accessions. We used multiple linear regression models based on non-standardized and standardized data from the middle part of the stem segments to evaluate which stem anatomical traits (predictive variables) best explain embolism resistance, with *P*_50_, *P*_12_ (air entry point) and *P*_88_ as response variables. Predictors were firstly selected based on biological knowledge, followed by a collinearity analysis through pairwise scatterplots and the variance inflation factor (VIF). To deduce the most parsimonious multiple linear regression model, we applied the step function from the stats package (R Core Team, 2016; available in CRAN, https://cran.r-project.org) to remove the least predictive variables each time according to the Akaike information criterion (AIC). Robust fitting of linear models through iteratively reweighted least squares (IWLS) and MM estimation (M-estimation with Tukey’s bi-weight initialized by a specific S-estimator) was used to deal with the outliers and leverages.

In addition, to assess the relative importance of the remaining explanatory variables of *P*_50_, we calculated the relative importance of regressors in linear models. Pearson’s correlation analysis was applied to assess the correlation between the predictive variables and *P*_50_. We used R version 3.6.3 in R Studio version 1.2.5033 for all analyses. All the differences were considered significant when the *P*-value was <0.05.

## RESULTS

### *Xylem vulnerability to embolism amongst the* Arabidopsis *accessions*

**Fig. 1.**
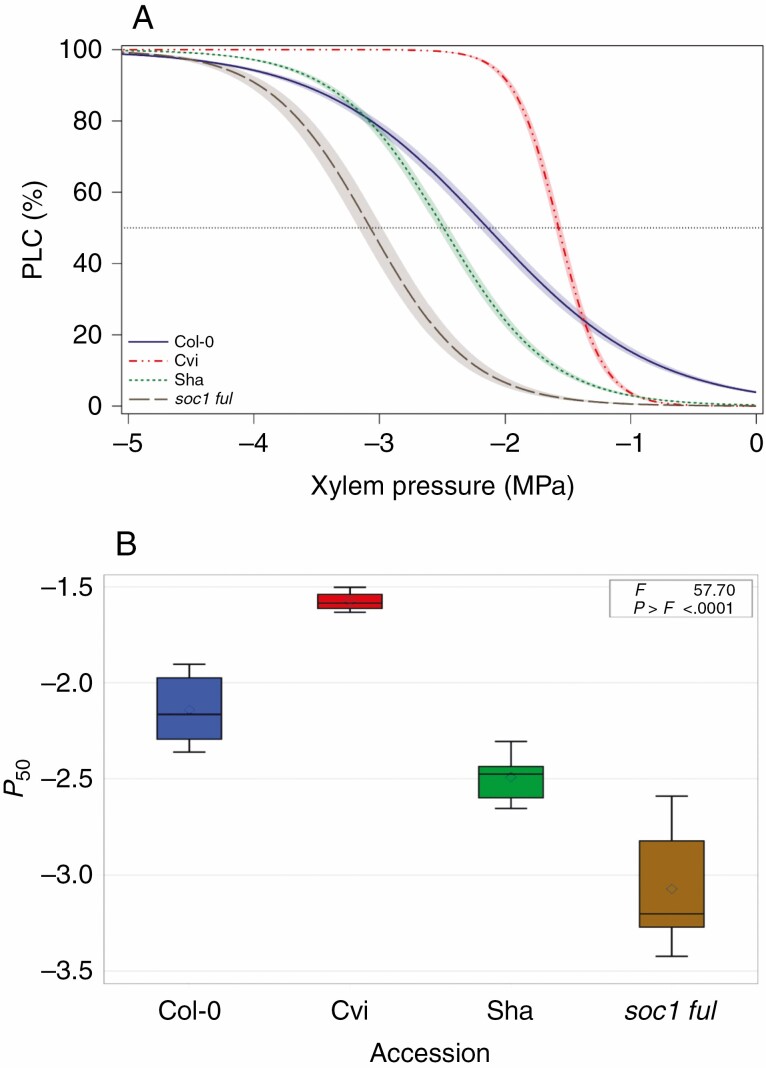
Stem *P*_*50*_ is significantly different across *A. thaliana* accessions. (A) Mean vulnerability curves for each accession presents the percentage loss of conductivity (PLC) as a function of xylem pressure (MPa). The dotted line shows 50 % loss of conductivity (*P*_50_). Shaded bands represent standard errors based on about ten vulnerability curves per accession. (B) Boxplot showing *P*_50_ distribution and variation within and between accessions (*P* = 0.05).

The *P*_50_ values of each of the accessions were significantly different from each other and varied 2-fold across the accessions studied (*F* = 57.70; *P* < 0.001) from −1.58 to −3.07 MPa (Fig. 1). Amongst the four accessions, stems of the *soc1 ful* double mutant were the most resistant to embolism [*P*_50_ = −3.07 ± 0.30 (s.d.) MPa; Supplementary Data Table S1] with a slope of 62 % MPa^−1^ (Fig. 1A), followed by Sha (*P*_50_ = −2.49 ± 0.11 MPa; slope = 59 % MPa^−s1^), Col-0 (*P*_50_ = −2.14 ± 0.18 MPa; slope = 38 % MPa^−1^) and Cvi (*P*_50_ = −1.58 ± 0.05 MPa; slope = 142 % MPa^−1^) (Supplementary Data Table S1; Fig. 1A). The *P*_50_ variation within accessions was remarkably low except for *soc1 ful*, in which it ranged from −2.59 to −3.42 MPa (Fig. 1B). Similar significant variation in *P*_12_ (*F* = 26.79; *P* < 0.001) was observed; for *P*_88_, Col-0 and Sha were not significantly different from each other (*F* = 34.8; *P* = 0.517).

### Stem anatomical traits amongst the accessions studied

**Fig. 2.**
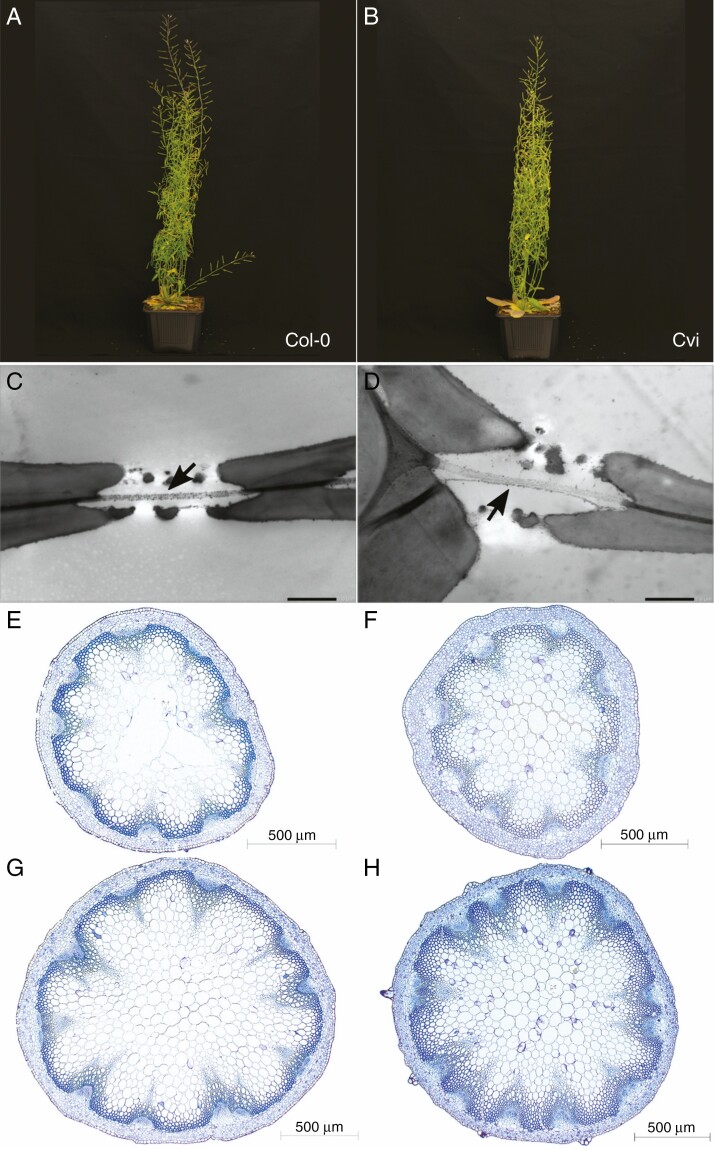
Growth form and cross-sections of inflorescence stems of Col-0 (left, 57 d after sowing) and Cvi (right, 57 d after sowing). (A, B) growth form. (C, D) TEM images of intervessel pit membranes (arrows). Scale bars = 1 μm. (E, F) LM images of cross-sections at the middle part of inflorescence stems. (G, H) LM images of cross-sections at the basal part of inflorescence stems.

**Fig. 3.**
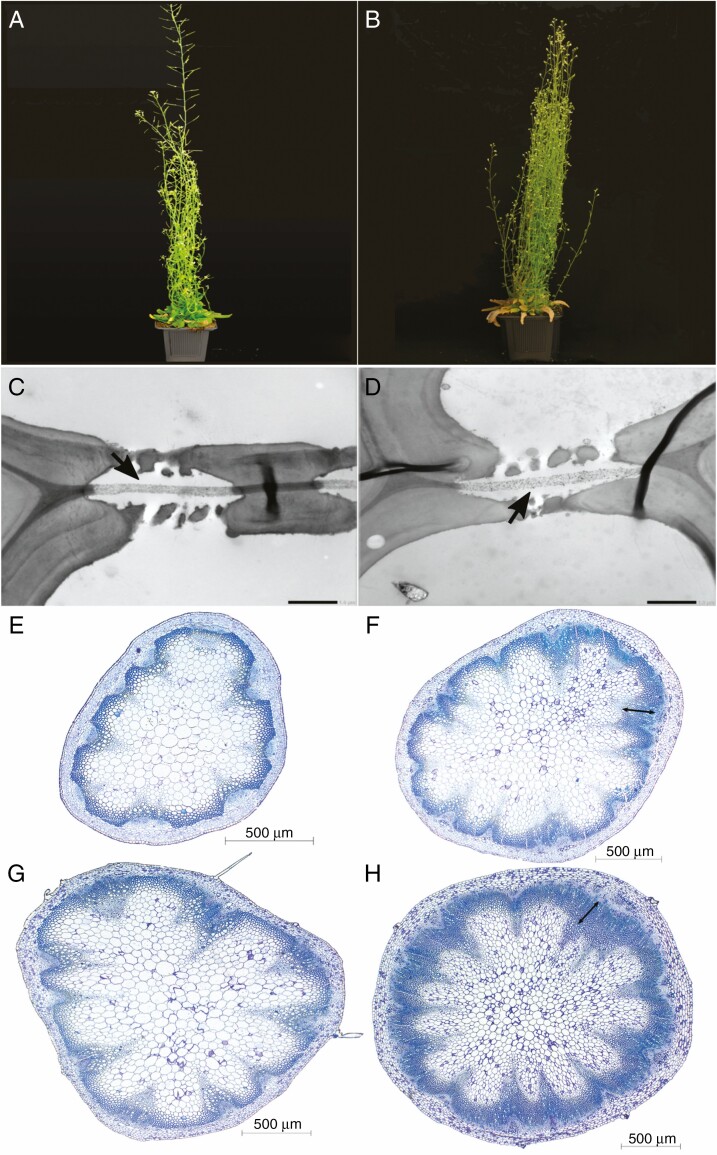
Growth form and cross-sections of inflorescence stems of Sha (left, 57 d after sowing) and *soc1 ful* (right, 80 d after sowing). (A, B) Growth form. (C, D) TEM images of intervessel pit membranes (arrows). Scale bars = 1 μm. (E, F) LM images of cross-sections at the middle part of inflorescence stems; the double-pointed arrow shows the wood cylinder. (G, H) LM images of cross-sections at the basal part of inflorescence stems; the double-pointed arrow shows the wood cylinder.

The features that were significantly different from each other among the accessions studied were intervessel pit membrane thickness (*T*_PM_) (*F* = 118.8; *P* < 2e^−16^; Supplementary Data Fig. S1a, Figs 2C, D and 3C, D), theoretical vessel implosion resistance (*T*_VW_/*D*_MAX_)^2^ (*F* = 37.35; *P* = 1.44e^−10^; Supplementary Data Fig. S1b) and proportion of fibre wall area per fibre cell area (*P*_FW_F_A_) (*F* = 65.33; *P* = 9.75e^−14^; Supplementary Data Fig. S1c). Meanwhile, the proportion of lignified area per total stem area (*P*_LIG_) of Col-0 was different from *soc1 ful* and Cvi (*F* = 18.68; *P* = 3.48e^−07^; Supplementary Data Fig. S1d), which was similar to Sha. Furthermore, the vessel grouping indexes (*V*_G_) of Col-0 and Cvi were similar, which was also the case for Sha and *soc1 ful; V*_G_ values of these two groups, however, were significantly different from each other (*F* = 43.45; *P* = 2.17e^−11^; Supplementary Data Fig. S1e). Vessel wall thicknesses (*T*_V_) of Col-0 and Cvi were different from each other, and different from Sha and *soc1 ful*, which had similar *T*_V_ values (*F* = 33.46; *P* = 5.52e^−10^; Supplementary Data Fig. S1f).

### Relationship between embolism resistance and anatomical features

**Fig. 4.**
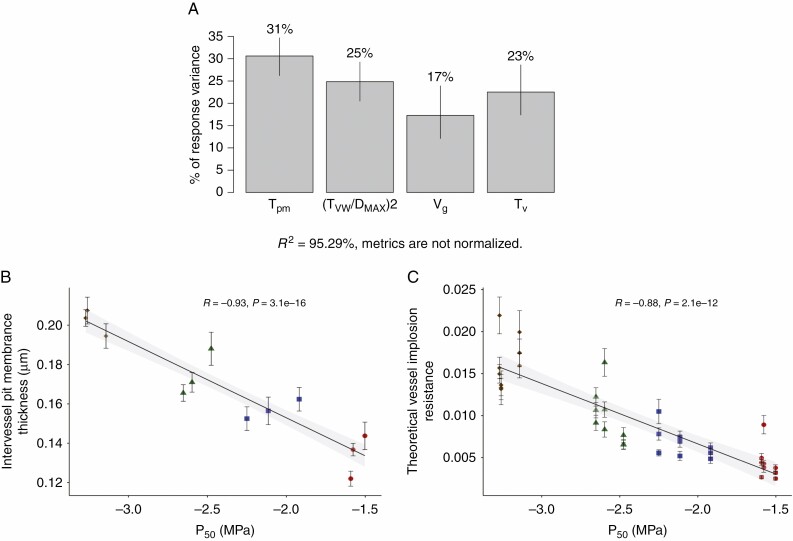
Relative importance and correlations of intervessel pit membrane thickness and theoretical vessel implosion resistance to P50, determined using the Lindemann, Merenda and Gold (LMG) method. (A) Relative importance of *P*_50_ variation is mainly explained by intervessel T_PM_ and theoretical vessel implosion resistance, (T_VW_/D_MAX_)^2^, based on *R*^2^ contribution averaged over orderings among regressors. (B) Negative correlation between thickness of intervessel pit membrane (T_PM_) and *P*_50_; (C) negative correlation between (T_VW_/D_MAX_)^2^ and *P*_50_. Colours and styles refer to the accession studied: Col-0, blue squares; Cvi, red circles; Sha, green triangles; *soc1 ful*, brown diamonds.

Both *T*_PM_ and (*T*_VW_/*D*_MAX_)^2^ strongly correlated positively with embolism resistance based on a Pearson correlation test (*r* = −0.93, *P* = 3.1e^−16^ and *r* = −0.88, *P* = 2.1e^−12^, respectively; Fig. 4B, C). Furthermore, there were correlations between embolism resistance and vessel wall thickness (*T*_V_) (*r* = −0.86, *P* = 2.5e^−11^; Supplementary Data Fig. S2a), between embolism resistance and vessel grouping index (*V*_G_) (*r* = −0.77, *P* = 3.6e^−08^; Supplementary Data Fig. S2b), between embolism resistance and proportion of lignified area per total stem area (*P*_LIG_) (*r* = −0.67, *P* = 7.2e^−06^; Supplementary Data Fig. S2c) and between embolism resistance and proportion of fibre wall per fibre cell area (*P*_FW_F_A_) (*r* = −0.73, *P* = 3.4e^−07^; Supplementary Data Fig. S2d).

Multiple regression analysis with robust fitting showed that the best predictors explaining *P*_50_ variation were *T*_PM_ (Figs 2C, D and 3C, D) and theoretical vessel implosion resistance ((*T*_VW_/*D*_MAX_)^2^), followed by vessel wall thickness (*T*_V_) and vessel grouping index (*V*_G_) (*R*^2^ = 0.9468 *P* < 2.2e^−16^) (Table 2). However, only *T*_PM_ and (*T*_VW_/*D*_MAX_)^2^ were highly significant in this model (*P* < 0.01) (Table 2). According to the regressor analysis, the relative importance of *T*_PM_ and (*T*_VW_/*D*_MAX_)^2^ in explaining *P*_50_ variation was 31 % and 25 %, respectively (Table 2, Fig. 4A). The proportion of lignified area per total stem area (*P*_LIG_) did not explain embolism resistance based on the most parsimonious multiple regression model (AIC score = −134.39; Table 2, Supplementary Data Table S2), but was included in the second most parsimonious model (AIC = −132.44; Supplementary Data Table S3).

**Table 2.** The best multiple regression model, based on AIC scores, of anatomical features explaining *P*_50_ variation in stems of the four *A. thaliana* accessions studied

Predictor	Estimate	Standard error	*z* value	Pr (>|*z*|)
(Intercept)	0.901	0.315	2.858	0.004262
*T*_PM_	−10.896	2.035	−5.356	8.522E−08***
(*T*_VW_/*D*_MAX_)^2^	−35.174	10.927	−3.219	0.001287**
*T*_V_	−0.516	0.239	−2.163	0.031*
*V*_G_	−0.280	0.183	−1.529	0.126

*** *P* < 0.001; ** *P* < 0.01; *P < 0.05

Correspondingly, *T*_PM_ also best explained *P*_12_ and *P*_88_ variations based on multiple regression models, followed by pit chamber depth (*D*_PC_) (*R*^2^ = 0.9507 *P* < 2.2e^−16^ and *R*^2^ = 0.8646 *P* < 3.88e^−13^, respectively) (Supplementary Data Tables S4 and S5). In addition, theoretical vessel implosion resistance ((*T*_VW_/*D*_MAX_)^2^) was included in the *P*_88_ multiple regression model (*P* < 0.05) (Supplementary Data Table S5), while *T*_V_ was included in the *P*_12_ multiple regression model as a significant predictor (*P* < 0.001) (Supplementary Data Table S4).

Correlations between the anatomical variables were as follows: thickness of intervessel pit membrane was strongly correlated to theoretical vessel implosion resistance <(*T*_VW_/*D*_MAX_)^2^>, vessel wall thickness <*T*_V_>, vessel grouping <*V*_G_> and proportion of fibre wall per fibre cell area <*P*_FW_F_A_> (*r* = 0.77, 0.76, 0.72 and 0.68, respectively; *P* < 0.001) (Supplementary Data Fig. S3). Apart from that, (*T*_VW_/*D*_MAX_)^2^ correlated with *T*_V_, *V*_G_ and *P*_FW_F_A_ (*r* = 0.77, 0.62 and 0.59, *P* < 0.001), *V*_G_ was correlated with *T*_V_ (*r* = 0.63; *P* < 0.001) (Supplementary Data Fig. S3) and the proportion of lignified area per total stem area (*P*_LIG_) showed correlations with *T*_PM_, *V*_G_, (*T*_VW_/*D*_MAX_)^2^ and *T*_V_ (*r* = 0.67, 0.66, 0.58 and 0.58, respectively; *P* < 0.001; Supplementary Data Fig. S3).

## DISCUSSION

We found 2-fold variation in stem *P*_50_ amongst the *A. thaliana* accessions studied (ranging from −1.5 to −3.0 MPa; Fig. 1), which was significantly associated with an increase in the thickness of the intervessel pit membrane (*T*_PM_; Fig. 4) and is in line with the air-seeding hypothesis. Our findings confirm earlier reports that *Arabidopsis* inflorescence stems with increased levels of lignification are better able to avoid drought-induced embolism than stems that are less lignified (Figs 2 and 3), which is based on (1) a more elaborate set of wild-type accessions (three versus one), (2) multiple vulnerability curves per accession compared with only one vulnerability curve per accession, and (3) more detailed anatomical observations compared with previous structure–function papers in *Arabidopsis* (Lens *et al.*, 2013; Tixier *et al.*, 2013). We investigated correlations amongst a range of anatomical traits related to stem lignification and uncovered statistical associations between increased lignification and *T*_PM_ and between vessel wall thickness (*T*_V_) and *T*_PM_. Our comparative approach suggests an indirect link between traits related to mechanical strength in stems and *P*_50_, with *T*_PM_ serving as the missing functional link between stem reinforcement and vulnerability to embolism.

### *Variation in stem P*_*50*_ *amongst* Arabidopsis *accessions agrees with other herbs and is best explained by intervessel T*_*PM*_

Our embolism resistance measurements with the Cavitron technique support earlier papers reporting values for the same species based on the more traditional centrifuge technique in combination with a portable water flow device (XYL’EM) (from −2.25 to −3.5 MPa; Lens *et al.*, 2013; Tixier *et al.*, 2013). Our data also fall within the range of the published *P*_50_ values for herbaceous eudicot species (Tyree *et al.*, 1986; Stiller and Sperry, 2002; Li *et al.*, 2009; Saha *et al.*, 2009; Rosenthal *et al.*, 2010; Nolf *et al.*, 2014; Skelton *et al.*, 2017; Dória *et al*., 2018, 2019; Bourbia *et al.*, 2020), although more negative *P*_50_ values (up to −7.5 MPa) of herbaceous stems, especially in grasses, have been reported in some papers (Lens *et al.*, 2016; Volaire *et al.*, 2018).

Amongst the anatomical traits we observed, *T*_PM_ strongly correlates with *P*_50_ and explains best the variation in *P*_50_ observed based on a statistical test showing the relative importance of regressors in our most parsimonious multiple linear regression model (Table 2; Fig. 4A, B). Our observations in *Arabidopsis* fit well with other published data of woody and herbaceous species where properly fixed intervessel pit membranes have been measured in stems that were subjected to *P*_50_ measurements (Li *et al.*, 2016; Dória *et al*., 2018, 2019; Supplementary Data Fig. S4). Furthermore, intervessel pit membrane thickness is the only trait that is also significant in the *P*_12_ and *P*_88_ multiple regression models, which emphasizes the functional relevance of *T*_PM_ in our dataset (Supplementary Data Tables S4 and S5). As highlighted before, this *T*_PM_–*P*_50_ correlation is undoubtedly functionally relevant because it fits nicely with the air-seeding mechanism. Although we do not fully understand exactly how this mechanism works at the ultrastructural level, the oversimplified 2-D view suggesting that air-seeding occurs via the single largest pit membrane pore should be abandoned (Wheeler *et al.*, 2005). Instead, a more realistic 3-D structure of intervessel pit membranes shows that a single pit membrane pore, being highly interconnected with other pores, has multiple constrictions that are often narrower than 50 or 20 nm when pit membranes are thinner or thicker than 300 nm, respectively (Zhang *et al.*, 2020). In other words, the chance of having a smaller pore constriction becomes higher with thicker pit membranes as this elongates the multiconstriction pit membrane pore. Consequently, air-seeding is not determined by the single largest pore in a pit membrane, but by the minimum constriction across all the interconnected pores in a given pit membrane (Kaack *et al.*, 2019; Zhang *et al.*, 2020). This explains why species with thicker intervessel pit membranes are better able to withstand air bubble spread between adjacent conduits under drought conditions than species with thinner intervessel pit membranes (Jansen *et al.*, 2009; Li *et al.*, 2016; Dória *et al.*, 2018). However, more ultrastructural observations of intact pit membranes and the role of surface-active substances such as phospholipids in the xylem sap and pit membranes should be carried out to improve our understanding of air bubble formation and spread at the ultrastructural level (Schenk *et al*., 2017, 2018; Zhang *et al.*, 2020).

### Disentangling the correlation between traits impacting mechanical strength and embolism resistance

Based on Pearson’s correlation test, the proportion of lignified area per total stem area (*P*_LIG_) is significantly correlated to *P*_50_ (Supplementary Data Figs S2c and S3). This is in line with our previous results in *Arabidopsis* (Lens *et al.*, 2013), in other lineages of Brassicaceae and Asteraceae (Dória *et al*., 2018, 2019) and in grasses (Lens *et al.*, 2016), showing that more woody/lignified stems are more resistant to embolism formation compared with close relatives with less woody/lignified stems. However, *P*_LIG_ is not included in the most parsimonious multiple regression *P*_50_ model (Table 2); it is retained in the second most parsimonious model (Supplementary Data Table S3), though, explaining only 10 % of the *P*_50_ variation (results not shown). Consequently, in our dataset, *P*_LIG_ is not a key functional trait contributing to vulnerability to embolism in stems of the *Arabidopsis* accessions studied. Still, it does have predictive value due to its correlation with other traits that are considered to be more relevant.

Interestingly, *P*_LIG_ is significantly correlated to several other lignification traits, of which intervessel pit membrane thickness (*T*_PM_), theoretical vessel implosion resistance (*T*_VW_/*D*_MAX_)^2^ and vessel wall thickness (*T*_V_) are prime examples (Supplementary Data Fig. S3). These three traits together explain 79 % of the *P*_50_ variation in the most parsimonious multiple regression model (Fig. 4A). When comparing the three multiple regression models for *P*_12_, P_50_ and *P*_88_ it is interesting to note that the depth of the pit chamber (*D*_PC_) is absent in the *P*_50_ model (Table 2) but pops up as highly significant in both the *P*_12_ and the *P*_88_ model (Supplementary Data Tables S4 and S5). It is hypothesized that shallower pit chambers minimize interconduit pit membrane stretching during aspiration and thereby reduce the mechanical stresses on the membranes in both angiosperms and gymnosperms (Hacke and Jansen, 2009; Lens *et al.*, 2011). However, *D*_PC_ does not seem to be generally correlated with embolism resistance across all lineages observed (Dória *et al.*, 2018).

The (indirect) correlation between *P*_50_ and traits impacting mechanical strength has also been highlighted in other studies that have found links between embolism resistance and the thickness-to-span ratio of conduits (Hacke *et al.*, 2001; Bouche *et al.*, 2014), vessel wall thickness (Jansen *et al.*, 2009; Li *et al.*, 2016; see also next paragraph), wood density (Jacobsen *et al.*, 2005; Hoffmann *et al.*, 2011; Anderegg *et al.*, 2016; Gleason *et al.*, 2016), fibre wall thickness (Jacobsen *et al*., 2005, 2007), lignin content (Pereira *et al.*, 2018) and lignin composition (Awad *et al.*, 2012; Lima *et al.*, 2018). Out of all these lignification characters, vessel wall reinforcement for a given lumen area, expressed either as thickness-to-span ratio of vessels or theoretical vessel implosion resistance, explains 25 % of the *P*_50_ variation (Fig. 4A), but only 3 % of the *P*_88_ variation (results not shown), and could potentially present a secondary functional link due to its direct association with long-distance water flow in plants, which is prone to negative pressures. Also, in conifers, the pressure causing conduit implosion is correlated with embolism resistance, but it is more negative than *P*_50_ for most species. Since vessel collapse due to negative pressures has never been observed in woody or herbaceous stems, it suggests that embolism occurs before the critical vessel implosion threshold is reached (Choat *et al.*, 2012; Bouche *et al.*, 2014), which is likely also the case for herbaceous species. There are only a few reports of (reversible) vessel collapse in the smallest leaf veins, which could be a mechanism to prevent embolism upstream in the major veins (Zhang *et al.*, 2016).

Variation in theoretical vessel implosion resistance ((*T*_VW_/*D*_MAX_)^2^) among the *A. thaliana* stems studied is mainly determined by the changes in vessel wall thickness (*T*_V_), explaining 64 % of the variation, whereas the maximum vessel lumen diameter (*D*_MAX_) only accounts for 31 % (Supplementary Fig. S5). This result is in line with Bouche *et al.* (2014), who found that *T*_V_ drives the variation in *T*_VW_/*D*_MAX_, suggesting that species tend to mechanically reinforce their conduits by increasing wall thickness instead of reducing conduit size in order to maintain a minimum level of hydraulic conductance. But at the same time *T*_V_ also positively correlates with *T*_PM_ (Supplementary Data Fig. S3), with thicker vessel walls leading to thicker intervessel pit membranes (Jansen *et al.*, 2009) and thus higher embolism resistance [*T*_V_ explaining 23 % of the *P*_50_ variation (Fig. 4A) and 18 % of the *P*_12_ variation (results not shown)]. On the other hand, other studies investigating the driver for *T*_VW_/*D*_MAX_ variation found that *D*_MAX_ is more important (Pittermann *et al.*, 2006; Sperry *et al.*, 2006), thereby reducing the relevance of conduit wall thickening.

Vessel grouping (*V*_G_), the final anatomical variable in the multiple regression *P*_50_ model, is the only character independent of lignification, and only accounts for 17 % of the variation (Fig. 4A) and 5 % of the *P*_88_ variation. Pearson’s correlation analysis shows a significant positive correlation between *V*_G_ and embolism resistance. Increased vessel connectivity safeguards all pathways in the 3-D vessel network when only one vessel in a vessel multiple is embolized (Carlquist, 1984; Lens *et al.*, 2011). This can only work when the intervessel pit membranes are sufficiently thick to isolate the embolisms in a given vessel multiple at a normal drought stress level, which seems to be the case in *Arabidopsis*. If *T*_PM_ is too thin, greater vessel connectivity increases the probability of embolism spreading via air-seeding, potentially leading to lethal levels of hydraulic failure (Tyree and Zimmermann, 2002; Loepfe *et al.*, 2007; Johnson *et al.*, 2020).

In conclusion, we found a 2-fold difference in stem *P*_50_ across the *Arabidopsis* accessions studied, with the woody mutant (*soc1 ful*) being most resistant to embolism compared with the wild-type accessions. This confirms earlier studies that found a link between increased stem lignification and increased embolism resistance in *Arabidopsis* and other lineages. However, a higher degree of stem lignification cannot functionally explain the pattern observed, and therefore stem lignification has to co-evolve with traits that functionally impact *P*_50_. Intervessel pit membrane thickness (*T*_PM_) and to a lesser extent theoretical vessel implosion resistance ((*T*_VW_/*D*_MAX_)^2^), vessel wall thickness (*T*_V_) and pit chamber depth (*D*_PC_), are strongly correlated with vulnerability to embolism and contribute most to the *P*_12_, *P*_50_ and *P*_88_ variation observed, making *T*_PM_ the main functional missing link between stem lignification and embolism resistance. Adding more accessions and performing complementary measurements related to drought tolerance in stems, leaves and roots will undoubtedly shed more light on the complex mechanism that this short-lived, herbaceous model species has developed in order to cope with periods of water shortage.

## SUPPLEMENTARY DATA

Supplementary data are available online at https://academic.oup.com/aob and consist of the following. Table S1: *P*_50_ and anatomical traits measured in the four *A. thaliana* accessions. Table S2: the most parsimonious multiple regression model with standardized data on anatomical features explaining *P*_50_ variation in stems of the four *A. thaliana* accessions studied. Table S3: the second most parsimonious multiple regression model of anatomical features explaining *P*_50_ variation in stems of the four *A. thaliana* accessions studied, including proportion of lignified area per total stem area. Table S4: the best multiple regression model, based on AIC scores, of anatomical features explaining *P*_12_ variation in stems of the four *A. thaliana* accessions studied. Table S5: the best multiple regression model, based on AIC scores, of anatomical features explaining *P*_88_ variation in stems of the four *A. thaliana* accessions studied. Figure S1: boxplots showing anatomical variation within and between accessions. Figure S2: scatter plots with regression lines showing the relationships between anatomical characters and *P*_50_. Figure S3: pairwise scatter plots based on Pearson’s correlation analysis showing the correlations of *P*_50_ and each stem anatomical trait studied and between all the predictive variables. Figure S4: scatter plot with regression line showing the relationship between *P*_50_ and intervessel pit membrane thickness of the woody and herbaceous angiosperms. Figure S5: relative importance of theoretical vessel implosion resistance variation and the relationship of theoretical vessel implosion resistance with vessel wall thickness and maximum vessel lumen diameter.

mcaa196_suppl_Supplementary_Figure_S1

mcaa196_suppl_Supplementary_Figure_S2

mcaa196_suppl_Supplementary_Figure_S3

mcaa196_suppl_Supplementary_Figure_S4

mcaa196_suppl_Supplementary_Figure_S5

mcaa196_suppl_Supplementary_Figure_Legends

mcaa196_suppl_Supplementary_Table_S1

mcaa196_suppl_Supplementary_Table_S2

mcaa196_suppl_Supplementary_Table_S3

mcaa196_suppl_Supplementary_Table_S4

mcaa196_suppl_Supplementary_Table_S5
