# Supplementary figures and images for: Intervessel pit membrane thickness best explains variation in embolism resistance amongst stems of Arabidopsis thaliana accessions
Source: Ann Bot. 2020 Nov 20;128(2):171–82. doi: 10.1093/aob/mcaa196 (PMC8324034; doi:10.1093/aob/mcaa196)

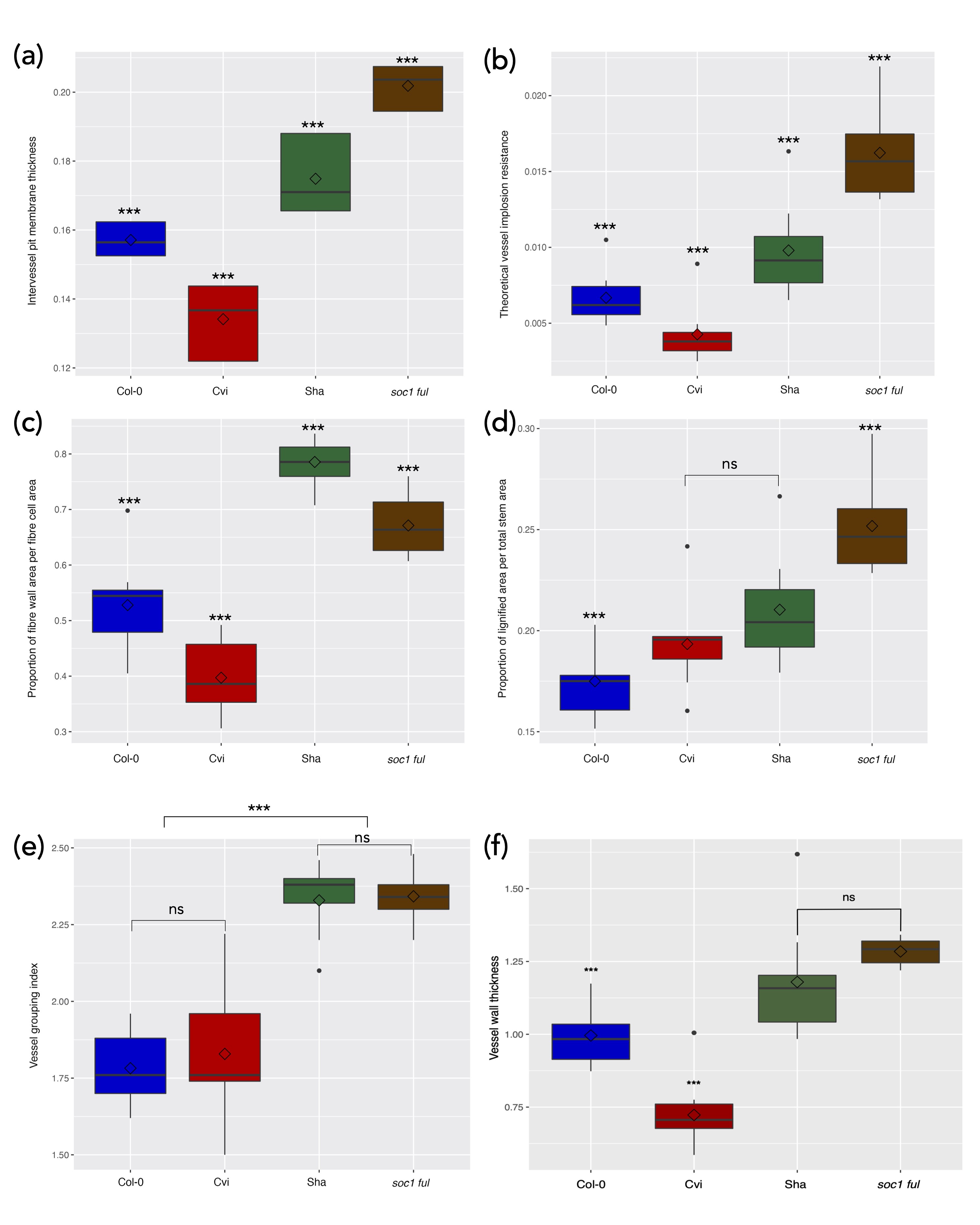

Supplement: mcaa196_suppl_Supplementary_Figure_S1 [file mcaa196_suppl_supplementary_figure_s1.jpeg]

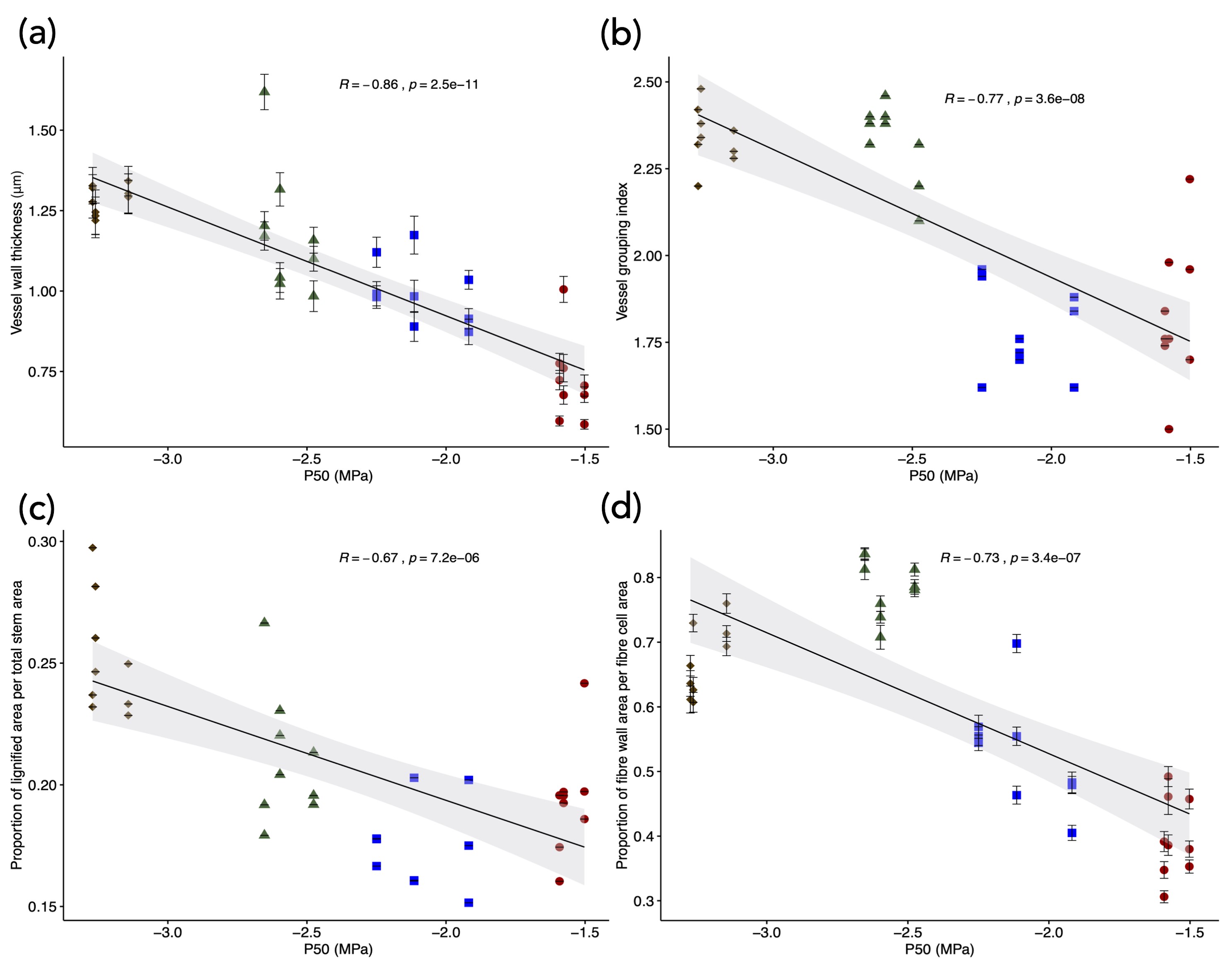

Supplement: mcaa196_suppl_Supplementary_Figure_S2 [file mcaa196_suppl_supplementary_figure_s2.jpeg]

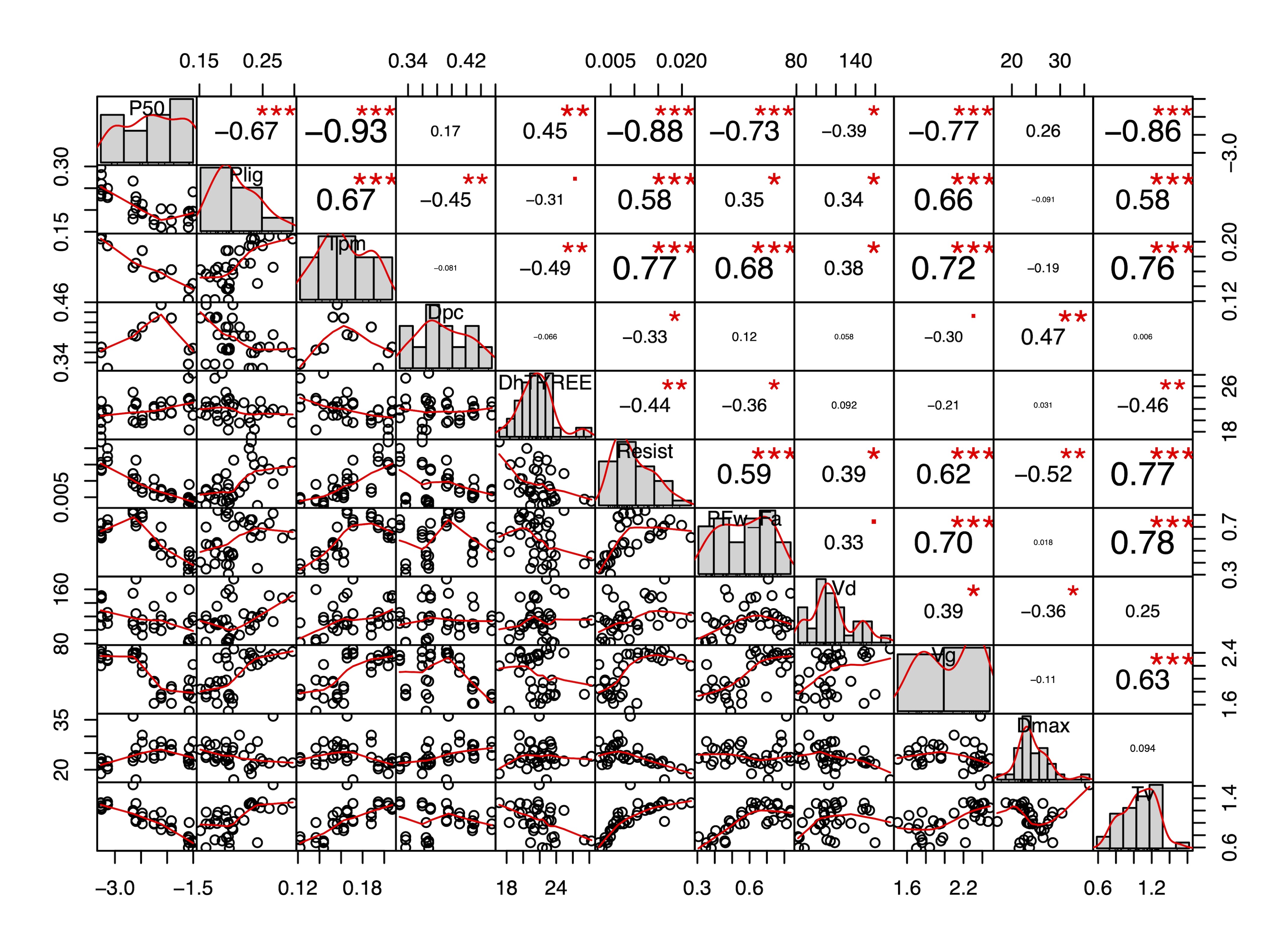

Supplement: mcaa196_suppl_Supplementary_Figure_S3 [file mcaa196_suppl_supplementary_figure_s3.jpeg]

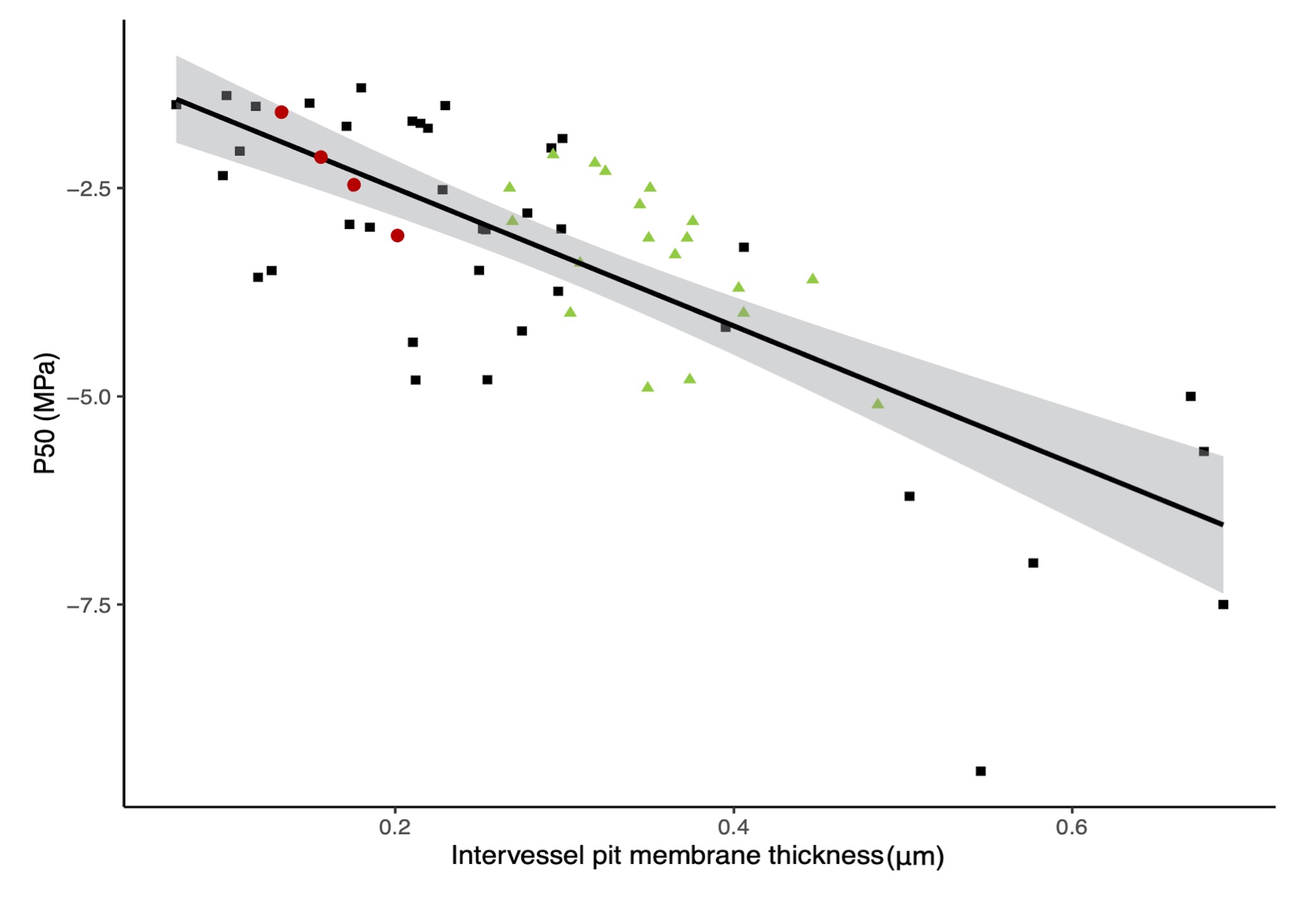

Supplement: mcaa196_suppl_Supplementary_Figure_S4 [file mcaa196_suppl_supplementary_figure_s4.jpeg]

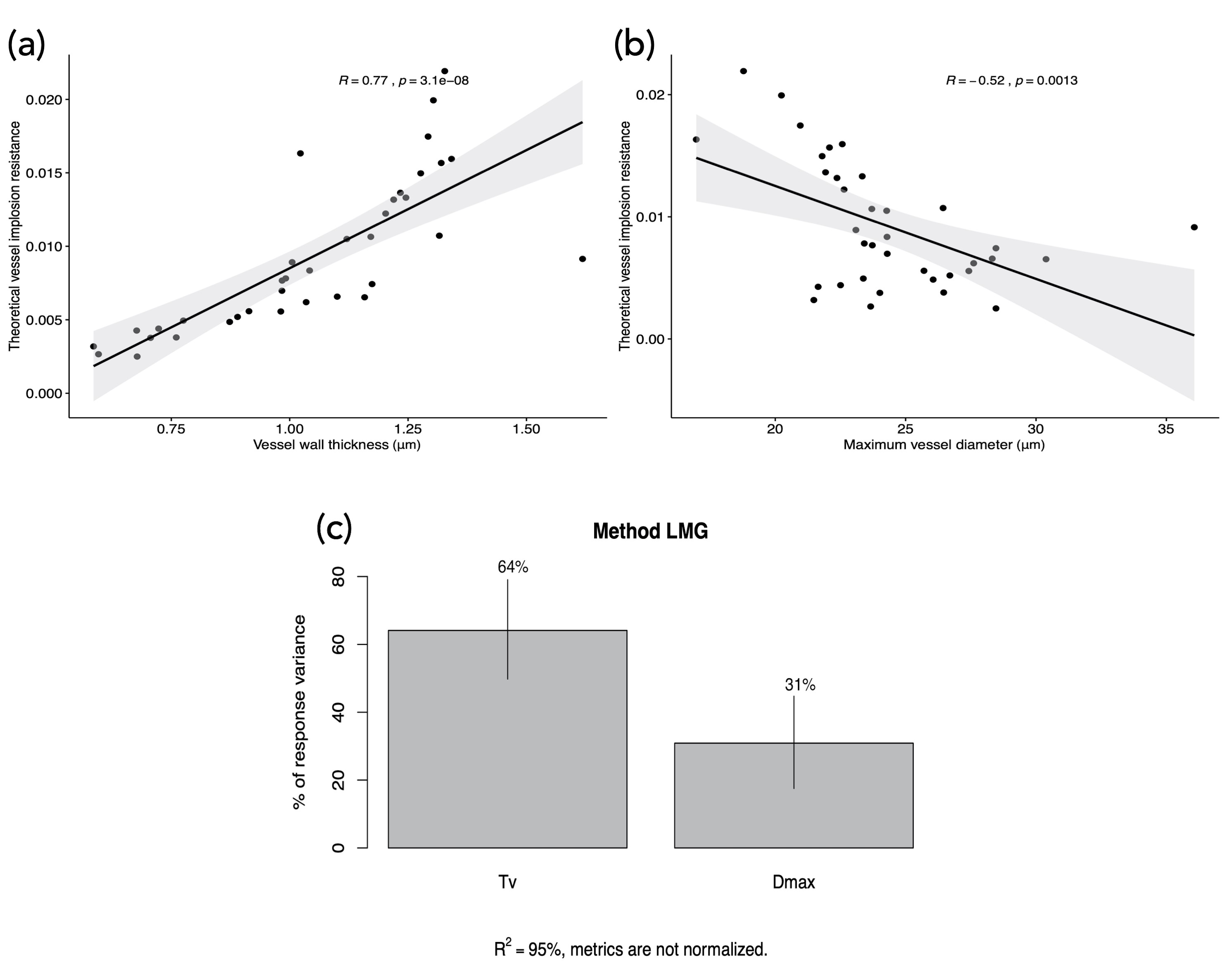

Supplement: mcaa196_suppl_Supplementary_Figure_S5 [file mcaa196_suppl_supplementary_figure_s5.jpeg]
