# Supplementary material for: Intervessel pit membrane thickness best explains variation in embolism resistance amongst stems of Arabidopsis thaliana accessions
Source: Ann Bot. 2020 Nov 20;128(2):171–82. doi: 10.1093/aob/mcaa196 (PMC8324034; doi:10.1093/aob/mcaa196)
Supplement: mcaa196_suppl_Supplementary_Figure_Legends [file mcaa196_suppl_supplementary_figure_legends.docx]

**Supplementary Figure captions**

**FIG. S1** Boxplots showing anatomical variation within and between accessions. (a) boxplot of intervessel pit membrane thickness (T_PM_); (b) boxplot of theoretical vessel implosion resistance (T_VW_/D_MAX_)^2^); (c) boxplot of proportion of fibre wall area per fibre cell area (PF_W_F_A_); (d) boxplot of proportion of lignified area per total stem area (P_LIG_); (e) boxplot of vessel grouping index (V_G_); ns = p-value > 0.05; *** p-value < 0.01

**FIG. S2** Scatter plots with regression lines showing the relationships between anatomical characters and *P*_50_. (a) the negative correlation between vessel grouping index (V_G_) and *P*_50_; (b) negative correlation between proportion of fibre wall area per fibre cell area (PF_W_F_A_) and *P*_50_; (c) negative correlation between proportion of lignified area per total stem area (P_LIG_) and *P*_50_. Colours and styles refer to the accession studied: Col-0 (blue-filled square), Cvi (red-filled circle), Sha (green-filled triangle) and *soc1 ful* (brown-filled diamond).

**FIG. S3** The pairwise scatter plots based on Pearson’s correlation analysis showing the correlations of *P*_50_ (response variable) and each stem anatomical trait studied (predictive variables) and between all the predictive variables comprising proportion of lignified area per total stem area (P_LIG_), intervessel pit membrane thickness (T_PM_), pit chamber depth (D_PC_), hydraulically weighted vessel diameter (D_H_ or D_H_TYREE), theoretical vessel implosion resistance ((T_VW_/D_MAX_)^2^ or Resist), proportion of fibre wall area per fibre cell area (PF_W_F_A_), vessel density (V_D_), vessel grouping index (V_G_), maximum vessel lumen diameter (D_MAX_)., and vessel wall thickness (T_V_) *** p-value < 0.01; ** p-value<0.01; * p-value < 0.05.

FIG. S4 Scatter plot with regression line showing the relationship between *P*_50_ and intervessel pit membrane thickness (T_PM_) of published woody and herbaceous angiosperms from Li *et al.* (2016; woody species marked as black-filled squares), Dória *et al.* (2018, 2019; mostly herbaceous species marked as green-filled triangles), and this study (red-filled circles).

FIG S5 Scatter plots with regression lines showing the relationship between (a) theoretical vessel implosion resistance ((T_VW_/D_MAX_)^2^) and vessel wall thickness (T_V_), and (b) theoretical vessel implosion resistance ((T_VW_/D_MAX_)^2^) and maximum vessel lumen diameter (D_MAX_); (c) the relative importance of theoretical vessel implosion resistance variation is mainly explained by vessel wall thickness (T_V_) based on R^2^ contribution averaged over orderings among regressors.
