# Supplementary material for: Intervessel pit membrane thickness best explains variation in embolism resistance amongst stems of Arabidopsis thaliana accessions
Source: Ann Bot. 2020 Nov 20;128(2):171–82. doi: 10.1093/aob/mcaa196 (PMC8324034; doi:10.1093/aob/mcaa196)
Supplement: mcaa196_suppl_Supplementary_Table_S1 [file mcaa196_suppl_supplementary_table_s1.doc]

**Table S1** *P*_50_ and anatomical traits measured (mean±SD) of the four *Arabidopsis thaliana* accessions.

| **Traits/Accessions** | **Col-0** | **Cvi** | **Sha** | ***soc1 ful*** |
| --- | --- | --- | --- | --- |
| ***P*_50_** | -2.14 ± 0.18 | -1.58 ± 0.05 | -2.49 ± 0.11 | -3.07 ± 0.30 |
| **P_LIG_  (middle part of stem)** | 0.175±0.001 | 0.193±0.016 | 0.210±0.009 | 0.252±0.013 |
| **T_PM_ (μm)** | 0.157±0.005 | 0.134±0.011 | 0.175±0.012 | 0.202±0.007 |
| **D_PC_ (μm)** | 0.438±0.015 | 0.357±0.017 | 0.400±0.010 | 0.353±0.022 |
| **D (μm)** | 20.954±0.154 | 22.577±1.202 | 20.714±1.211 | 20.036±2.155 |
| **D_H_ (μm)** | 21.995±0.189 | 23.456±1.384 | 21.403±.127 | 20.625±1.967 |
| **D_MAX_ (μm)** | 25.992±0.828 | 23.854±0.744 | 25.837±2.834 | 21.562±0.870 |
| **T_V_** | 0.996±0.048 | 0.723±0.082 | 1.179±0.133 | 1.284±0.045 |
| **(T_VW_/D_MAX_)^2^** | 0.007±0.001 | 0.004±0.001 | 0.010±0.003 | 0.016±0.002 |
| **PF_W_F_A_** | 0.528±0.063 | 0.397±0.049 | 0.786±0.047 | 0.671±.045 |
| **V_D_** | 116.444±5.251 | 102.706±9.753 | 120.756±14.931 | 127.756±10.735 |
| **V_G_** | 1.782±0.057 | 1.829±0.115 | 2.329±0.108 | 2.342±0.050 |

P_LIG_ = proportion of lignified area per total stem area; T_PM_ = intervessel pit membrane thickness; D_PC_ = pit chamber depth; D = vessel diameter; D_H_ = hydraulically weighted vessel diameter; D_MAX_ = maximum vessel diameter; T_V_ = vessel wall thickness; (T_VW_/D_MAX_)^2^ = theoretical vessel implosion resistance; P_FW_F_A_ = proportion of fibre wall area per fibre cell area; V_D_ = vessel density; V_G_ = vessel grouping index
