# Supplementary material for: Intervessel pit membrane thickness best explains variation in embolism resistance amongst stems of Arabidopsis thaliana accessions
Source: Ann Bot. 2020 Nov 20;128(2):171–82. doi: 10.1093/aob/mcaa196 (PMC8324034; doi:10.1093/aob/mcaa196)
Supplement: mcaa196_suppl_Supplementary_Table_S2 [file mcaa196_suppl_supplementary_table_s2.doc]

**Table S2** The most parsimonious multiple regression model with standardized data of anatomical features explaining *P*_50_ variation in stems of the four *Arabidopsis thaliana* accessions studied.

| **Predictors** | **Estimate** | **Std. Error** | **z value** | **Pr (>|z|)** |
| --- | --- | --- | --- | --- |
| **(Intercept)** | -1.237988 | 0.079647 | -15.5435 | < 2.2E-16 |
| **T_PM_** | -0.931430 | 0.173915 | -5.3557 | 8.524E-08*** |
| **(T_VW_/D_MAX_)^2^** | -0.683314 | 0.212280 | -3.2189 | 0.001287** |
| **T_V_** | -0.533092 | 0.246432 | -2.1632 | 0.030522* |
| **V_G_** | -0.274562 | 0.179581 | -1.5289 | 0.126288 |

T_PM_ = intervessel pit membrane thickness; (T_VW_/D_MAX_)^2^ = theoretical vessel implosion resistance; T_V_ = vessel wall thickness; V_G_ = vessel grouping index; *** p-value < 0.001; ** p-value < 0.01; * p-value < 0.05
