# Supplementary material for: Intervessel pit membrane thickness best explains variation in embolism resistance amongst stems of Arabidopsis thaliana accessions
Source: Ann Bot. 2020 Nov 20;128(2):171–82. doi: 10.1093/aob/mcaa196 (PMC8324034; doi:10.1093/aob/mcaa196)
Supplement: mcaa196_suppl_Supplementary_Table_S3 [file mcaa196_suppl_supplementary_table_s3.doc]

**Table S3** Second most parsimonious multiple regression model of anatomical features explaining *P*_50_ variation in stems of the four *Arabidopsis thaliana* accessions studied, including proportion of lignified area per total stem area (P_LIG_).

| **Predictors** | **Estimate** | **Std. Error** | **z value** | **Pr (>|z|)** |
| --- | --- | --- | --- | --- |
| **(Intercept)** | 0.902453 | 0.321147 | 2.81010 | 0.004953 |
| **T_PM_** | -10.94886 | 2.29588 | -4.76890 | 1.852E-06*** |
| **(T_VW_/D_MAX_)^2^** | -35.08942 | 11.41948 | -3.07280 | 0.002121** |
| **T_V_** | -0.51645 | 0.25839 | -1.99870 | 0.045636* |
| **V_G_** | -0.28617 | 0.19144 | -1.49480 | 0.134960 |
| **P_LIG_** | 0.09505 | 1.28027 | 0.07420 | 0.940816 |

T_PM_ = intervessel pit membrane thickness; (T_VW_/D_MAX_)^2^ = theoretical vessel implosion resistance; T_V_ = vessel wall thickness; V_G_ = vessel grouping index; P_LIG_ = proportion of lignified area per total stem area; *** p-value < 0.001; ** p-value < 0.01; *p-value < 0.05
