# Supplementary material for: Intervessel pit membrane thickness best explains variation in embolism resistance amongst stems of Arabidopsis thaliana accessions
Source: Ann Bot. 2020 Nov 20;128(2):171–82. doi: 10.1093/aob/mcaa196 (PMC8324034; doi:10.1093/aob/mcaa196)
Supplement: mcaa196_suppl_Supplementary_Table_S4 [file mcaa196_suppl_supplementary_table_s4.doc]

**Table S4** The best multiple regression model, based on AIC scores, of anatomical features, explaining *P*_12_ variation in stems of the four *Arabidopsis thaliana* accessions studied.

| **Predictors** | **Estimate** | **Std. Error** | **z value** | **Pr (>|z|)** |
| --- | --- | --- | --- | --- |
| **(Intercept)** | -1.84631 | 0.52630 | -3.50810 | 0.0004513 |
| **T_PM_** | -25.44504 | 2.57654 | -9.87560 | < 2.2E-16*** |
| **P_FW_F_A_** | 0.73844 | 0.52827 | 1.39790 | 0.1621552 |
| **T_V_** | -0.80998 | 0.22178 | -3.65220 | 0.0002600*** |
| **D_PC_** | 12.34489 | 1.17463 | 10.5096 | < 2. 2E-16*** |

T_PM_ = intervessel pit membrane thickness; P_FW_F_A_ = proportion of fibre wall area per fibre cell area; T_V_ = vessel wall thickness; D_PC_ = pit chamber depth; *** p-value < 0.001; ** p-value < 0.01; *p-value < 0.05
