# Supplementary material for: Intervessel pit membrane thickness best explains variation in embolism resistance amongst stems of Arabidopsis thaliana accessions
Source: Ann Bot. 2020 Nov 20;128(2):171–82. doi: 10.1093/aob/mcaa196 (PMC8324034; doi:10.1093/aob/mcaa196)
Supplement: mcaa196_suppl_Supplementary_Table_S5 [file mcaa196_suppl_supplementary_table_s5.doc]

**Table S5** The best multiple regression model, based on AIC scores, of anatomical features, explaining *P*_88_ variation in stems of the four *Arabidopsis thaliana* accessions studied.

| **Predictors** | **Estimate** | **Std. Error** | **z value** | **Pr (>|z|)** |
| --- | --- | --- | --- | --- |
| **(Intercept)** | 4.16481 | 0.67485 | 6.17150 | 6.764E-10*** |
| **T_PM_** | 11.37418 | 3.08554 | 3.68630 | 0.0002276*** |
| **(T_VW_/D_MAX_)^2^** | -42.08524 | 16.36473 | -2.57170 | 0.0101199* |
| **P_FW_F_A_** | -1.24828 | 0.62798 | -1.98780 | 0.0468351* |
| **V_G_** | -0.77311 | 0.46031 | -1.67950 | 0.0930509 |
| **D_PC_** | -15.82159 | 1.66181 | -9.52070 | < 2.2E-16*** |

T_PM_ = intervessel pit membrane thickness; (T_VW_/D_MAX_)^2^ = theoretical vessel implosion resistance; P_FW_F_A_ = proportion of fibre wall area per fibre cell area; V_G_ = vessel grouping index; D_PC_ = pit chamber depth; *** p-value < 0.001; ** p-value < 0.01; *p-value < 0.05
